# Cognition of abdominal pain and abdominal discomfort in Chinese patients with irritable bowel syndrome with diarrhea

**DOI:** 10.1186/s13030-023-00286-1

**Published:** 2023-09-08

**Authors:** Jia Lu, Yang Chen, Lili Shi, Xiaoqing Li, Guijun Fei, Ji Li, Aiming Yang, Xiucai Fang

**Affiliations:** 1 Department of Gastroenterology, Peking Union Medical College Hospital, Chinese Academy of Medical Sciences and Peking Union Medical College, 1# Shuaifuyuan, Dongcheng District, Beijing, 100730 China; 2 Department of Gastroenterology, Henan Provincial People’s Hospital, People’s Hospital of Zhengzhou University, School of Clinical Medicine, Henan University, Zhengzhou, Henan China; 3 Department of Psychological Medicine, Peking Union Medical College Hospital, Chinese Academy of Medical Sciences and Peking Union Medical College, Beijing, China

**Keywords:** Irritable bowel syndrome with diarrhea, Abdominal pain, Abdominal discomfort, Cognition

## Abstract

**Background:**

In Asia, the proportion of patients with irritable bowel syndrome (IBS) with abdominal discomfort alone is significantly higher than that in western countries. The purposes of this study are to understand the cognition of abdominal pain and abdominal discomfort in Chinese patients with IBS and to compare the clinical characteristics of patients with abdominal pain alone and with abdominal discomfort alone.

**Methods:**

Patients with IBS with diarrhea (IBS-D) who met the Rome III diagnostic criteria and had episodes of at least one day/week were consecutively enrolled. The cognition of abdominal pain and abdominal discomfort were investigated through face-to-face unstructured interview. Patients were divided into a pain group and a discomfort group according to the cognition interviews, then the characteristics and severity of symptoms (IBS symptom severity scale, IBS-SSS), IBS quality of life (IBS-QOL) and psychological state were compared between groups.

**Results:**

A total of 88 patients with IBS-D were enrolled. Most of the patients with self-reported abdominal pain described their pain as spasm/cramping; patients with self-reported abdominal discomfort had as many as 24 different descriptions of discomfort. Most patients having abdominal pain and discomfort could accurately distinguish the two symptoms. The degree of abdominal pain in the pain group was higher than abdominal discomfort in the discomfort group (*P* = 0.002). There was no significant difference in IBS-SSS, extra-intestinal pain, IBS-QOL, and psychological state between the two groups.

**Conclusions:**

For Chinese patients with IBS-D, abdominal pain and abdominal discomfort are two different symptoms, but they have similar clinical features.

**Trial registration:**

ChiCTR, ChiCTR1900028082. Registered 11 December 2019 - Retrospectively registered, http://www.chictr.org.cn.

## Background

Irritable bowel syndrome (IBS) is a common functional bowel disorder, and IBS with diarrhea (IBS-D) is the most common subtype in China, accounting for 74.1% of IBS [1]. The diagnosis of IBS is based on symptoms: abdominal pain and abdominal discomfort associated with altered defecation are the core symptoms of IBS using Rome III criteria [2]. However, the Rome IV criteria eliminated “abdominal discomfort” and adjusts the frequency of symptoms, leading to a decrease in the global prevalence of IBS from 10.1% to 4.1% [3]. The revision of the Rome IV criteria was mainly based on studies of western populations. Abdominal discomfort is very common symptom in IBS patients in Asia [4]. IBS patients in China and Thailand who met Rome III but did not fulfill Rome IV criteria mainly reported only abdominal discomfort without pain, accounting for 22.6%-84.2% of all Rome III-IBS patients [5–7], which is much higher than the data reported in western countries (2.4%-9.9%) [8, 9]. A Rome Foundation Asian working team reported that abdominal discomfort is second to abdominal pain as a bothersome symptom (18.2% vs 19.5%) in patients with functional bowel disease (of which 45.1% is IBS) in 11 Asian cities [10]. Thus, the deletion of “discomfort” from Rome IV criteria has a greater impact on the diagnosis and treatment of IBS patients in Asia.

The cognition of the symptom spectrum of abdominal pain and abdominal discomfort affects the symptom reporting of patients. In the Rome III definition, abdominal discomfort means “an uncomfortable sensation not described as pain” [2]. It is unclear whether the symptom spectrum and pathophysiological mechanisms of discomfort are essentially different from pain. Cultural and linguistic differences have been noted in IBS patients' cognition of abdominal discomfort [11]. An early study carried out in the United States found that discomfort encompasses a range of symptoms such as bloating, gas, sensation of incomplete evacuation, and urgency for some patients. But compared with abdominal discomfort, IBS patients have a more consistent cognition of abdominal pain, which is often described as pain or spasm/cramping [12]. In Asian populations, there is still a lack of cognition study to compare the difference between abdominal pain and abdominal discomfort in IBS patients. This study aimed to investigate the cognition of abdominal pain and abdominal discomfort in Chinese IBS-D patients and to provide reference for understanding the particularity of symptoms and for further pathophysiological research on Asian IBS patients.

## Methods

### Participants

Patients with IBS-D who met the Rome III criteria and had episodes at least one day per week were consecutively enrolled from the Gastroenterology Clinics of Peking Union Medical College Hospital (PUMCH) from January 2019 to January 2022. Patients with organic gastrointestinal diseases, connective tissue diseases, and metabolic diseases confirmed by laboratory and endoscopy examinations in the last year were excluded. Considering that psychological abnormalities may affect a patients' perception and cognition of symptoms, patients with moderate to severe anxiety/depression assessed by Hamilton Anxiety Scale (HAMA) and Hamilton Depression Scale (HAMD) were also excluded. This study was approved by the Ethics Committee of PUMCH (JS-1753). All subjects gave their written, informed consent before inclusion. The study was registered at China Clinical Trials Registration Center (ChiCTR1900028082).

### Measures

#### Individual cognitive interviews

A questionnaire was developed with reference to the survey of abdominal pain and abdominal discomfort and the cognitive interview of IBS patients in the United States [12, 13], combined with the clinical experiences of functional gastroenterology specialists in the Department of Gastroenterology of PUMCH. A professionally trained investigator interviewed all patients in a one-to-one, face-to-face unstructured interview.

The cognitive interview process is as follows. First, inform the purpose of the interview, then ask patients to describe in detail the symptoms of abdominal pain/abdominal discomfort they experienced. Next ask if abdominal pain/abdominal discomfort is associated with defecation. After that, explain to the patient in detail the definitions of “abdominal pain”, “abdominal discomfort” and “associated with defecation”. Abdominal pain means an unpleasant feeling and emotional experience caused by actual or potential tissue injury in the abdomen: it can also occur with other symptoms. Abdominal discomfort means a subjective and unpleasant feeling not described as pain or bloating, distension, flatulence, urgency, difficulty in defecation, incomplete emptying, or anorectal pain, but it can also occur at the same time with the above symptoms [14]. Associated with defecation means abdominal pain/abdominal discomfort occurs or aggravates before, during or after defecation, which can be relieved gradually after defecation.

After explanation, patients are asked to confirm whether they have definite abdominal pain/abdominal discomfort associated with defecation; and patients with both abdominal pain and abdominal discomfort confirm whether abdominal pain and abdominal discomfort are the same symptoms. Then they are asked whether abdominal pain and abdominal discomfort occur at the same time, what is the difference, and whether they can accurately distinguish between the two symptoms. The location, degree, frequency, duration, and effect on IBS quality of life (IBS-QOL) of abdominal pain/discomfort reported by the patients were recorded. The degree of abdominal pain/discomfort associated with defecation was evaluated by visual analogue scale (VAS) based on the recorded symptoms over the most recent three months.

Patients were divided into two groups according to the confirmed symptoms of defecation-associated abdominal pain/discomfort after the cognitive interviews. Pain group: patients have defecation-associated abdominal pain only, without abdominal discomfort. Discomfort group: patients have defecation-associated abdominal discomfort only, without abdominal pain. The main study purpose of this section is to compare abdominal pain and abdominal discomfort, we did not further evaluate and compare the clinical data of patients with both abdominal pain and abdominal discomfort.

#### Gastrointestinal symptoms and extra-intestinal pain assessment

After the cognitive interviews, gastrointestinal (GI) symptoms were assessed using the IBS symptom questionnaire, including demographic information, main bowel symptoms, and extra-intestinal pain. The frequency of symptom episodes was recorded as four grades: 1 day/week, 2–3 days/week, 4–6 days/week, and daily. The duration of symptom episodes was recorded as five grades: < 10 min/day, 10-30 min/day, 30-60 min/day, > 60 min/day, and persistently. The stool form was scored by the Bristol Stool Form Scale. The severity of IBS symptoms was evaluated by the IBS symptom severity scale (IBS-SSS) [15].

#### IBS-QOL assessment

The simplified Chinese version of IBS-QOL compiled by Huang et al*. *according to Patrick was used to evaluate the quality of life of patients [16, 17].

#### Psychological evaluation

Psychological state was assessed by HAMA and HAMD through conversation and observation by a professionally trained inestigator [18, 19].

#### Statistical analysis

Data were analyzed using SPSS 23.0 software (IBM Corporation, Armonk, NY, USA). Continuous variables and categorical variables were presented as mean ± standard deviation or median (interquartile range [IQR]) and ratio, respectively. Continuous normal distribution variables were analyzed by independent two-sample *t*-test, categorical variables by nonparametric test (Mann–Whitney U test), and chi-square test was used for comparison between ratios. A *P*-value of < 0.05 was considered statistically significant.

## Results

### Subjects

In total, 88 patients with IBS-D were enrolled (55 male; 62.5%). Their mean age was 39.6 ± 10.45 years. The mean disease course of IBS-D was 6.5 (IQR [4.0, 14.3]) years.

### Cognition of abdominal pain and abdominal discomfort

Before the interviewer explained the definitions of abdominal pain and abdominal discomfort, a total of 67 patients reported abdominal pain, of whom 44 (65.7%) described it as colic, spasm/cramping, twisting, or throbbing. Of 15 patients who described abdominal pain as dull pain, 4 reported it as swelling pain, 2 as bearing-down pain, 2 as ache, 4 as burning pain, and 1 as stuffy pain. Among them, 5 had multiple descriptions of abdominal pain.

A total of 50 patients reported abdominal discomfort before the explanation, of whom 23 (46%) described abdominal discomfort as indescribable, unspeakable uncomfort of the abdomen. Six patients (12%) described abdominal discomfort as pain or mild pain, and these patients also reported abdominal pain at the same time. Five patients (10%) described abdominal discomfort as bloating, flatulence, or sensation of defecation. A few patients described abdominal discomfort as sore, sour, cool, needling, abdominal hardening, burning, carsickness, emptiness, tightness, unrelaxing, numbness, or presence of abdomen.

After the interviewer explained the definitions of abdominal pain, abdominal discomfort, and associated with defecation, 67 patients with self-reported abdominal pain confirmed that they actually had abdominal pain, and it was clear that abdominal pain was associated with defecation. Of the 50 cases of self-reported abdominal discomfort, 36 (72%) confirmed that they actually had defecation-associated abdominal discomfort.

A total of 15 patients reported both defecation-associated abdominal pain and abdominal discomfort. After the explanation of the definitions, 14 patients (93.3%) believed that abdominal pain and abdominal discomfort were different types of symptoms and they could distinguish two symptoms, and 12 patients thought that abdominal pain and abdominal discomfort would cause different functional damage. The majority (13/15) believed that abdominal pain had a greater impact on life.

### Clinical characteristics of abdominal pain vs abdominal discomfort

The patients were divided into a Pain Group (*n* = 52, 27 male, 25 female) and a Discomfort Group (*n* = 21, 17 male, 4 female) according to abdominal pain/discomfort associated with defecation confirmed after cognitive interviews. There were no significant differences in age, body mass index (BMI), labor type, education level, and economic status between groups. The percentage of male patients in the discomfort group was significantly higher than that in the pain group (81.0% vs 51.9%, χ^2^ = 5.265, *P* = 0.022) (Table 1).

**Table 1:** Demographic data of IBS-D patients with abdominal pain alone vs abdominal discomfort alone

Variable	Pain group (*n* = 52)	Discomfort group (*n* = 21)	*t* / χ^2^-value	*P*-value
Male, %	27 (51.9)	17 (81.0)	5.265	0.022
Age in years	40.1 ± 10.89	39.7 ± 9.82	0.157	0.876
BMI in kg/m^2^	23.6 ± 3.94	23.5 ± 2.96	0.097	0.923
Physical labor, %	13 (25.0)	5 (23.8)	0.011	0.915
Education Level, college and above, %	33 (63.5)	13 (61.9)	0.941	0.625
Family economic status, well-off & above, %	41 (78.8)	17 (81.0)	0.041	0.840

Data presented as number (%) or mean ± SD

*IBS-D* Irritable bowel syndrome with diarrhea, *BMI* Body mass index, *SD* Standard deviation

#### Location of abdominal pain vs abdominal discomfort

**Fig. 1:**
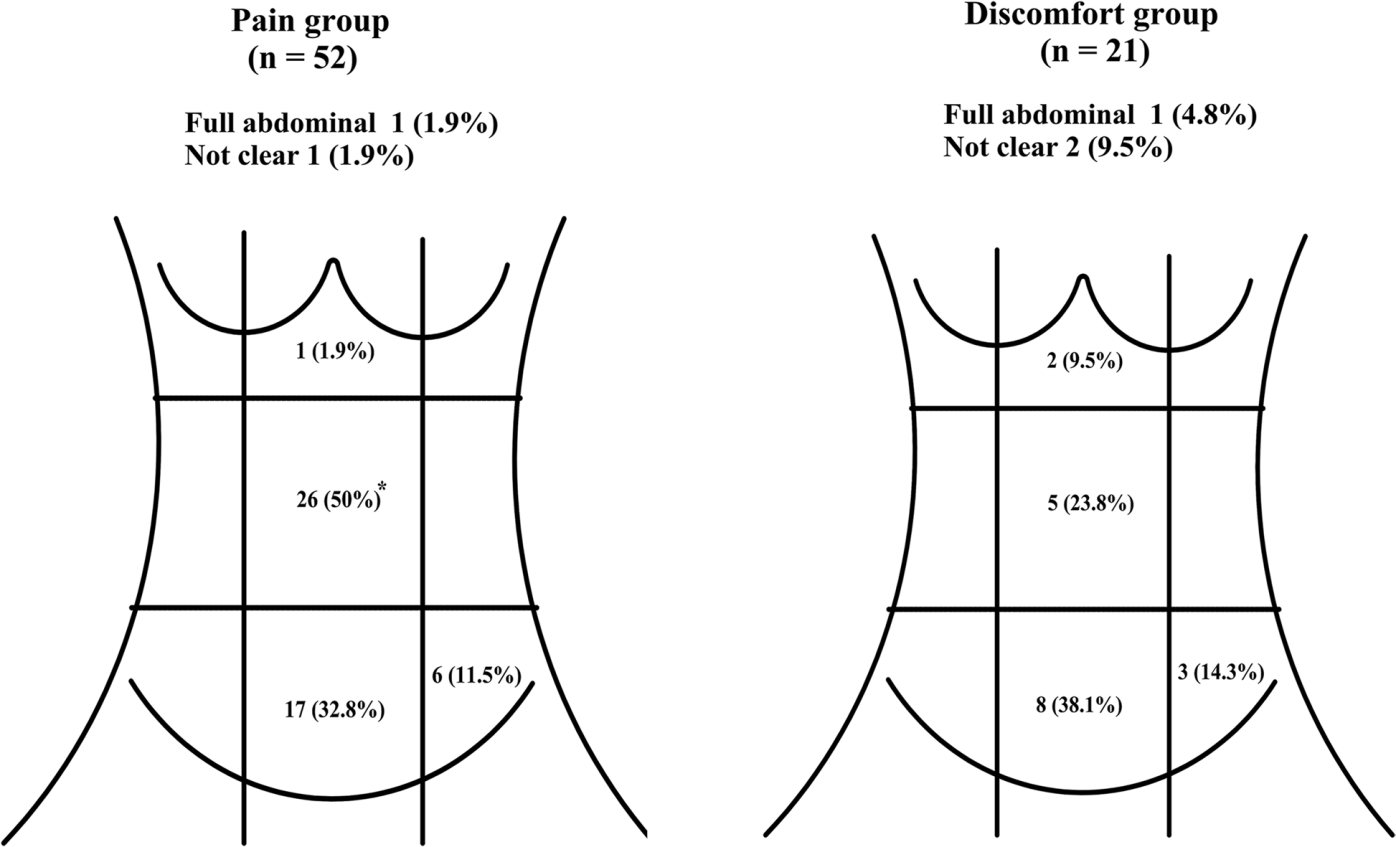
Location of abdominal pain and abdominal discomfort in patients with IBS-D for the pain and discomfort groups. Abdominal pain /discomfort is more located around the navel in the abdominal pain group than in the abdominal discomfort group (χ^2^ = 4.200, *P* = 0.04). Numbers (percentage) are shown in figures. *P < 0.05. IBS-D, irritable bowel syndrome with diarrhea

The most common locations of abdominal pain/discomfort were periumbilical and lower abdomen. The percentage of patients with periumbilical symptoms in the pain group was higher than that in the discomfort group (χ^2^ = 4.200, *P* = 0.040). Abdominal discomfort was located in the upper abdomen in 9.5% of patients, and 9.5% were “unclear” about the location of symptoms in the discomfort group (Fig. 1).

#### Characteristics and severity of GI symptoms

**Fig. 2:**
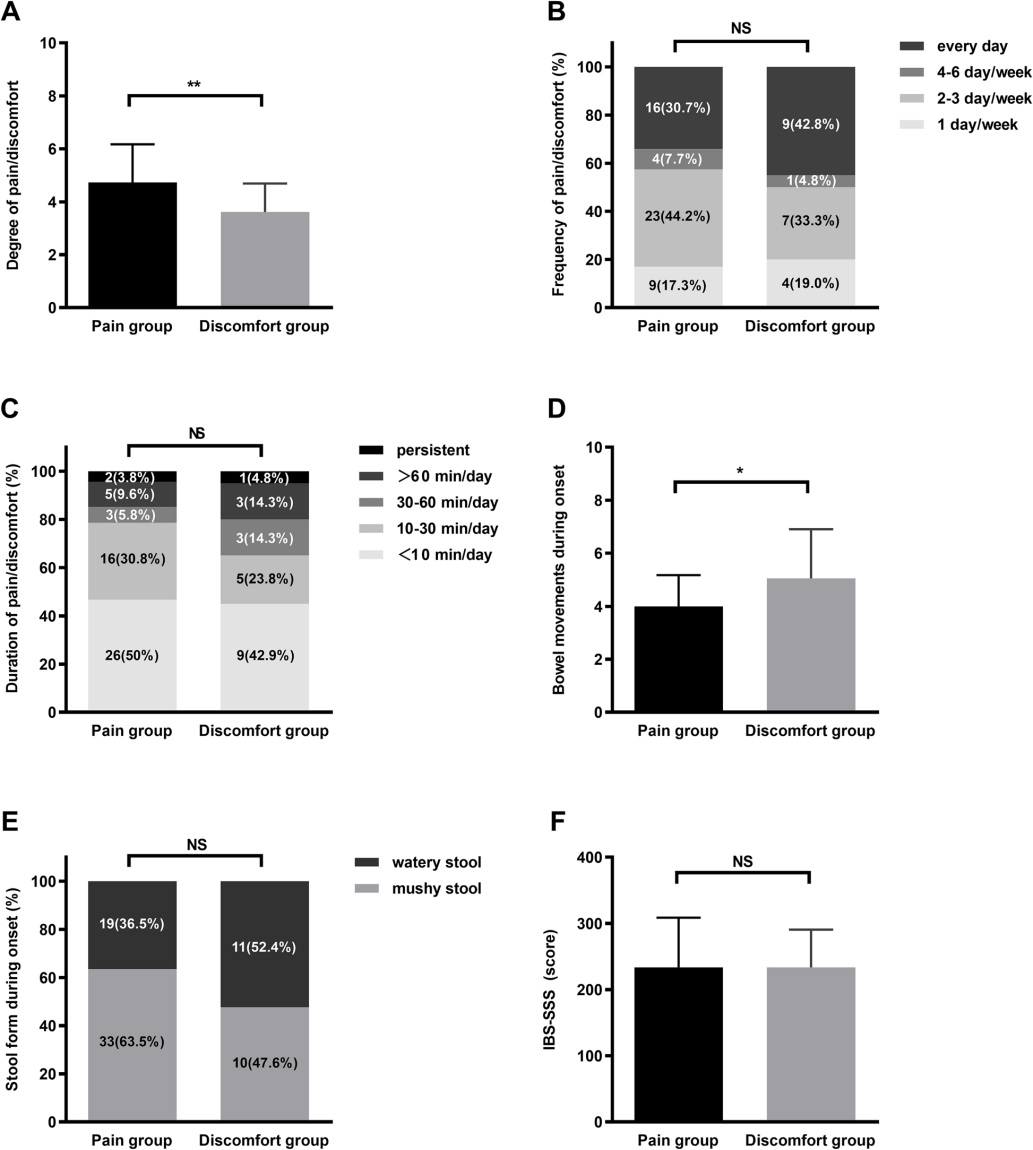
Clinical characteristics of IBS-D patients in the pain and discomfort groups. **A** The degree of abdominal pain in the abdominal pain group was more severe than the degree of discomfort in the abdominal discomfort group (*t* = 3.188, *P* = 0.002). **B** and **C** No significant differences were noted in frequency or duration of abdominal pain/discomfort between the two groups (*P* > 0.05). **D** The number of bowel movements during symptom onset in the discomfort group was significantly higher than that in the pain group (*t* = -2.400, *P* = 0.024). **E** and **F** No significant differences were noted in stool form during symptom onset or IBS-SSS score between the two groups (*P* > 0.05). Numbers (percentage) are shown in figures **B**, **C**, **E**. **P < 0.01, *P < 0.05. IBS-D, irritable bowel syndrome with diarrhea; IBS-SSS, IBS symptom severity scale; NS, not significant

The degree of abdominal pain in the pain group was more sever than that for abdominal discomfort in the discomfort group (4.73 ± 1.4 vs 3.62 ± 1.0, *t* = 3.188, *P* = 0.002) (Fig. 2A). There was no significant difference in the frequency or duration of defecation-associated abdominal pain/discomfort between the two groups (χ^2^ = 1.978, *P* = 0.740; χ^2^ = 2.059, *P* = 0.725) (Fig. 2B, C). The number of bowel movements during symptom onset in the discomfort group was significantly higher than that in the pain group (5.04 ± 1.85 vs 4.00 ± 1.17, *t* = -2.400, *P* = 0.024) (Fig. 2D), while there was no significant difference in the stool form during symptom onset between the two groups (χ^2^ = 1.551, *P* = 0.213) (Fig. 2E).

The IBS-SSS scores of the pain and discomfort groups were 233.5 ± 75.1 and 233.6 ± 57.0, respectively (*t* = -0.004, *P* = 0.997) (Fig. 2F).

#### Coexisting extra-intestinal pain

The proportion of patients with extra-intestinal pain in the pain and discomfort groups were 90.4% (47/52) and 76.2% (16/21), respectively (χ^2^ = 2.549, *P* = 0.110). There was no significant difference in the proportion of retrosternal pain, epigastric pain, headache, other pain (such as joint pain, backache), dyspareunia, or dysmenorrhea in female patients between the two groups (Table 2). It is worth noting that abdominal pain or abdominal discomfort during menstruation would be aggravated in all 13 female patients with dysmenorrhea (12 in the pain group and 1 in the discomfort group).

**Table 2:** Coexisting extra-intestinal pain in IBS-D patients with abdominal pain alone vs abdominal discomfort alone

Items	Pain group (*n* = 52)	Discomfort group (*n* = 21)	χ^2^-value	*P*-value
Retrosternal pain, %	5 (9.6)	2 (9.5)	0.000	0.990
Epigastric pain, %	13 (25.0)	5 (23.8)	0.011	0.915
Headache, %	24 (46.2)	10 (47.6)	0.013	0.910
Dyspareunia, %	7 (13.5)	2 (9.5)	0.215	0.643
Dysmenorrhea, %	12 (48.0)	1 (25.0)	1.757	0.185
Other pains, %	35 (67.3)	9 (42.9)	3.735	0.053

Data presented as number (%)

*IBS-D* Irritable bowel syndrome with diarrhea

### IBS-QOL

**Fig. 3:**
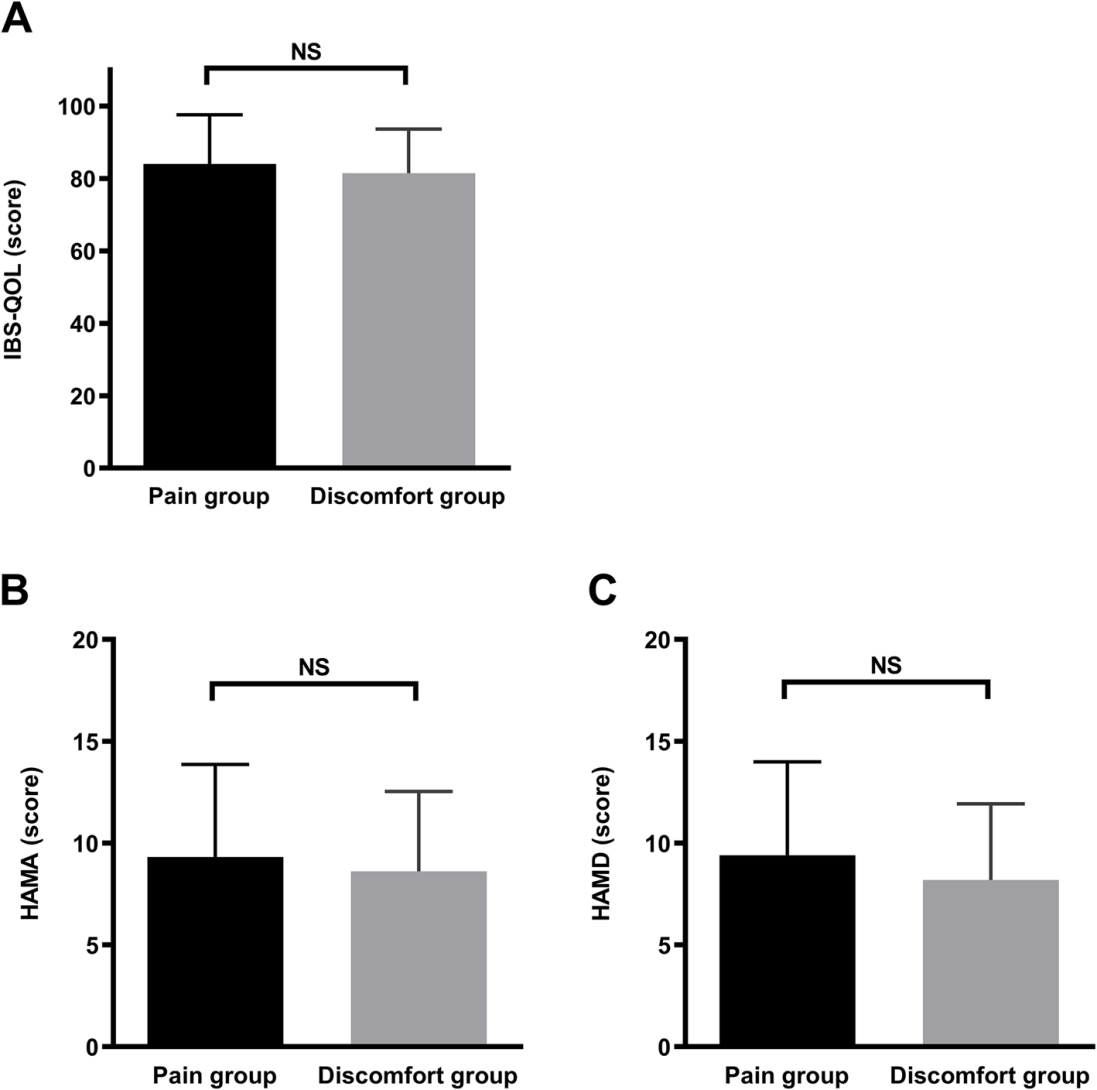
Comparison of IBS-QOL, HAMA, HAMD of patients with IBS-D of the pain and discomfort groups. No significant differences were noted in the IBS-QOL, HAMA, or HAMD scores of the two groups (*P* > 0.05). IBS-D, irritable bowel syndrome with diarrhea; IBS-QOL, IBS quality of life, HAMA, Hamilton Anxiety Scale; HAMD, Hamilton Depression Scale; NS, not significant

The IBS-QOL scores of the pain and discomfort groups were 84.0 ± 13.6 and 81.5 ± 12.2, respectively (*t* = 0.743, *P* = 0.460) (Fig. 3A).

### Psychological state

The HAMA scores of the pain and discomfort groups were 9.33 ± 4.5 and 8.62 ± 3.9, respectively (*t* = 0.626, *P* = 0.533) (Fig. 3B). The HAMD scores of the pain and discomfort groups were 9.40 ± 4.6 and 8.19 ± 3.7, respectively (*t* = 0.334, *P* = 0.286) (Fig. 3C).

## Discussion

This study found that Chinese IBS-D patients could accurately experience and report abdominal pain: most patients described abdominal pain as spasm/cramping, but the feelings and descriptions of abdominal discomfort were varied. Most patients were able to distinguish between abdominal discomfort and abdominal pain. The degree of abdominal pain was more sever than abdominal discomfort, but there was no significant difference in IBS-SSS, coexisting extra-intestinal pain, IBS-QOL, or psychological state between patients with abdominal pain and abdominal discomfort as the main symptom.

It is generally believed that abdominal pain is the most important symptom of IBS [20]. However, the Rome Foundation Asia Working Team reported that abdominal discomfort is the second most bothersome symptom in Asian patients with functional bowel disease, after only abdominal pain [10]. An online survey of patients with IBS with predominant constipation (IBS-C) conducted in a population study in Japan found that 64.3% of IBS-C patients had abdominal discomfort and 29.1% had abdominal pain and that 15.3% of the patients considered abdominal discomfort and 4.5% considered abdominal pain as the most bothersome symptom [21]. In addition, a study in the United States reported that the proportion of abdominal pain and bloating-type discomfort in IBS patients was similar (60% vs 66%): 60% of the patients listed bloating-type discomfort and 29% listed abdominal pain as the most bothersome symptom [22]. The severity of abdominal discomfort was independently correlated with QOL damage in IBS patients [13].

There is a lack of an accurate definition of abdominal discomfort at present [2]. Cultural background, socio-economic status, education level, and other factors will affect patients' feeling and expression of symptoms [23], which may be the main factors leading to the differences between results from eastern and western studies. Compared with diseases with clear biological basis, functional gastrointestinal diseases are more likely to be affected by cultural factors [24]. Even in the same cultural and linguistic environment, the spectrum of abdominal discomfort reported by IBS patients in America is very broad [12, 13]. For Chinese IBS-D patients, only asking about “abdominal discomfort” cannot obtain their accurate feelings. The descriptions of abdominal discomfort are varied, and the use of Chinese dialects makes the connotation of abdominal discomfort more complicated. Among the patients with self-reported abdominal discomfort, 28% considered mild pain, bloating, flatulence and sense of defecation as abdominal discomfort. In a multilingual environment, doctors need to have the ability to understand various descriptions of abdominal discomfort.

Chinese IBS patients can accurately experience and report abdominal pain. In our study, 65.7% of the patients described abdominal pain as spasm/cramping, and other descriptions were associated with pain, which is similar to American patients [12], indicating that the cognition, experience and description of the abdominal pain of IBS patients are relatively clear and consistent in different cultures. Some patients in the U.S. considered abdominal discomfort as being a kind of mild pain [11, 13]. Fang et al. compared the characteristics of abdominal pain and abdominal discomfort of Chinese patients by the use of the Rome III-IBS definitions. They found that the degree of abdominal pain and abdominal discomfort was moderate in most patients and that 22.3% of their patients with abdominal pain alone had severe abdominal pain, while 10.8% of their patients with abdominal discomfort alone had severe abdominal discomfort (*P*= 0.007). There was no significant difference in the frequency of abdominal pain/discomfort, number of bowel movements or stool form during symptom onset, extra-intestinal pain, depression or anxiety state, or quality of life between the two groups [7]. We found that the score of abdominal pain was higher than that of abdominal discomfort after cognitive interviews (4.73 ± 1.4 vs 3.62 ± 1.0), but both in moderate degree. Also, there was no significant difference in the frequency, duration of abdominal pain/discomfort, or IBS-SSS between the two groups. Thus, although the degree of abdominal discomfort is less severe than abdominal pain, patients with abdominal discomfort alone are not IBS with mild symptoms because of the overall severity and the effect of abdominal discomfort on quality of life.

According to reports, 13.9% ~ 29% of IBS patients have both abdominal pain and abdominal discomfort [7, 13]. In this study, 15 patients (17%) reported both abdominal pain and abdominal discomfort associated with defecation. After the explanation of the definitions of abdominal pain and abdominal discomfort, most patients could distinguish between the two. They understood that abdominal pain and abdominal discomfort would cause different functional damage and that abdominal pain had a greater impact on life. This shows that Chinese IBS patients can accurately distinguish between abdominal pain and abdominal discomfort, and the significant difference in the description of abdominal discomfort reflects the diversity and complexity of the pathophysiological mechanism of abdominal discomfort, to some extent.

Abdominal pain in patients with IBS is associated with visceral hypersensitivity caused by a variety of mechanisms, including diet, low-grade intestinal inflammation, changes in intestinal microbiota and psychological abnormality [25]. Spasm/cramping in patients with IBS is associated with intestinal spasmodic contraction [26]. Abdominal discomfort is more common in patients with IBS-C [21], Japanese scholars speculate that abdominal discomfort may be related to abnormal intestinal motility. At present, there is a lack of studies comparing differences in the pathophysiological mechanisms of abdominal pain and abdominal discomfort. Clinical analysis suggests that abdominal pain and abdominal discomfort are probably different experiences and feelings of similar stimuli [7, 13, 22]. Studies from the Netherlands [27] and Sweden [9] found that compared with IBS patients who fullfill the Rome III criteria, Rome IV-positive subjects were significantly more likely to be female and have greater pain severity. Meta analysis showed that female IBS patients were more likely to report abdominal pain than men, and both IBS and healthy women reported increased IBS symptoms during menses vs. other phases [28]. the anterior cingulate cortex (ACC) of the brain is import in pain processing, and the thickness of the bilateral subgenual anterior cingulate cortex (sgACC) in female but not male IBS patients became thinner and was correlated with the severity of abdominal pain [29]. In this study, most patients with abdominal discomfort alone were male, indicating that sex or sex hormones may affect the experience and report of symptoms, female patients may be more likely to report abdominal pain under similar stimuli. Our brain imaging study found that visceral hypersensitivity was more prominent in patients with abdominal discomfort alone and that the occurrence of discomfort was primarily related with the regions responsible for complex cognitive function and fear regulation (dorsal ACC) [30]. It is necessary to compare the pathophysiological mechanisms of abdominal pain and abdominal discomfort from the perspective of gut-brain interaction.

### The study limitations

IBS-D is the main subtype in China, other subtypes were not included in this study. Cases with both abdominal pain and abdominal discomfort were relatively few. There was only one interviewer in this study. She received professional psychological training for the cognitive interview to maintain as much objectivity and consistency as possible, It is unclear if this possibly biased the results. Considering the possible effects of depression and anxiety on symptom experience and report, patients with obvious psychological abnormalities were excluded, and the results could not reflect the condition of these patients.

## Conclusions

Abdominal pain and abdominal discomfort are two different symptoms for Chinese IBS-D patients, but they have similar clinical characteristics. Abdominal discomfort is one of the main symptoms of Asian IBS patients, which has a significant impact on disease severity and quality of life. Further comparative study of the pathophysiological mechanisms of abdominal pain and abdominal discomfort will be helpful to formulate diagnostic criteria and treatment strategies for IBS in Asian populations.

## Data Availability

We are not able to share our data because sharing data is not approved by our ethics committees.

## Abbreviations

IBS: Irritable bowel syndrome
IBS-D: IBS with diarrhea
IBS-SSS: IBS symptom severity scale
IBS-QOL: IBS quality of life
PUMCH: Peking Union Medical College Hospital
HAMA: Hamilton Anxiety Scale
HAMD: Hamilton Depression Scale
VAS: Visual analogue scale
GI: Gastrointestinal
IQR: Interquartile range
IBS-C: IBS with predominant constipation
ACC: Anterior cingulate cortex
SgACC: Subgenual anterior cingulate cortex
